# Structural synaptic elements are differentially regulated in superior temporal cortex of schizophrenia patients

**DOI:** 10.1007/s00406-012-0306-y

**Published:** 2012-03-23

**Authors:** Andrea Schmitt, Fernando Leonardi-Essmann, Pascal F. Durrenberger, Sven P. Wichert, Rainer Spanagel, Thomas Arzberger, Hans Kretzschmar, Mathias Zink, Mario Herrera-Marschitz, Richard Reynolds, Moritz J. Rossner, Peter Falkai, Peter J. Gebicke-Haerter

**Affiliations:** 1 Department of Psychiatry and Psychotherapy, University of Göttingen, von-Siebold-Str. 5, 37075 Göttingen, Germany; 2 Institute of Psychopharmacology, Central Institute of Mental Health, Medical Faculty Mannheim, University of Heidelberg, J5, 68159 Mannheim, Germany; 3 Human Disease Immunogenetics Group, Department of Infectious Diseases and Immunity, Imperial College Faculty of Medicine, Hammersmith Hospital, Commonwealth Building, Du Cane Road, London, W12 0NN UK; 4 Max-Planck Institute for Experimental Medicine, Hermann-Rein-Str.3, 37075 Göttingen, Germany; 5 Institute of Neuropathology, Ludwig-Maximilians-University of Munich, Feodor-Lynen-Strasse 23, 81377 Munich, Germany; 6 Department of Psychiatry, Central Institute of Mental Health, University of Heidelberg, J5, 68159 Mannheim, Germany; 7 Programme of Molecular and Clinical Pharmacology, ICBM, Medical Faculty, University of Chile, Av. Independencia 1027, Santiago 7, Chile; 8 Wolfson Neuroscience Laboratories, Imperial College Faculty of Medicine, Hammersmith Hospital Campus, Burlington Danes Building, Du Cane Road, London, W12 0NN UK; 9 Laboratory of Neuroscience (LIM27), Institute of Psychiatry, University of Sao Paulo, Rua Dr. Ovidio Pires de Campos 785, São Paulo, SP 05453-010 Brazil; 10 Department of Psychiatry, LMU Munich, Nußbaumstr. 7, 80336 Munich, Germany

**Keywords:** Schizophrenia, Superior temporal cortex, Cytoskeleton, Synaptic plasticity, Gene expression, Microarray

## Abstract

Inaccurate wiring and synaptic pathology appear to be major hallmarks of schizophrenia. A variety of gene products involved in synaptic neurotransmission and receptor signaling are differentially expressed in brains of schizophrenia patients. However, synaptic pathology may also develop by improper expression of intra- and extra-cellular structural elements weakening synaptic stability. Therefore, we have investigated transcription of these elements in the left superior temporal gyrus of 10 schizophrenia patients and 10 healthy controls by genome-wide microarrays (Illumina). Fourteen up-regulated and 22 downregulated genes encoding structural elements were chosen from the lists of differentially regulated genes for further qRT-PCR analysis. Almost all genes confirmed by this method were downregulated. Their gene products belonged to vesicle-associated proteins, that is, synaptotagmin 6 and syntaxin 12, to cytoskeletal proteins, like myosin 6, pleckstrin, or to proteins of the extracellular matrix, such as collagens, or laminin C3. Our results underline the pivotal roles of structural genes that control formation and stabilization of pre- and post-synaptic elements or influence axon guidance in schizophrenia. The glial origin of collagen or laminin highlights the close interrelationship between neurons and glial cells in establishment and maintenance of synaptic strength and plasticity. It is hypothesized that abnormal expression of these and related genes has a major impact on the pathophysiology of schizophrenia.

**Electronic supplementary material:**

The online version of this article (doi:10.1007/s00406-012-0306-y) contains supplementary material, which is available to authorized users.

## Introduction

Schizophrenia is regarded as a disorder with disrupted connectivity of neuronal networks involving multiple brain regions [14, 67]. Along these lines, subtle volume and fractional anisotropy reductions of the gray and white matter of the heteromodal association cortex including the superior temporal cortex (STG) have been reported in meta-analyses [13, 70, 84]. The left STG gray matter was smaller, and the left greater than right asymmetry was reduced in schizophrenia patients [1, 18, 26, 41]. However, despite these findings based on magnetic resonance imaging studies, the underlying alterations are widely unknown. In schizophrenia, altered synaptic plasticity and connectivity during neurodevelopment and adulthood have been suggested [20, 82]. On the molecular level, several lines of research have established contributions of the GABAergic and glutamatergic systems [52], and also of myelination-related events [23] to the pathophysiology of the disease. Comparing microarray analyses of different brain regions, the STG is among the most affected regions in schizophrenia [25]. A recent oligonucleotide-microarray study of the STG revealed altered expression of genes involved in neurotransmission and synaptic function [7]. Messenger RNAs of six synaptic proteins including SNAP-25, synaptotagmin, and syntaxin were increased in schizophrenia patients compared to control subjects [72]. On the protein level, complexins are reduced in the STG [12]. Since pre- and post-synaptic structural genes control remodeling of dendritic spines or maintain stability of the synaptic cytoskeleton, our attention has been drawn to genes involved in synaptic plasticity associated with extracellular and cytoskeletal structural elements in the left STG of schizophrenia patients. The turnover time of actin in dendritic spines is approximately 44 s [74], which highlights the dynamics of cytoskeletal elements in neuronal fiber endings. Moreover, structural elements of the extracellular matrix play pivotal roles in axon guidance, synapse formation, and stabilization. Proteins like collagen or laminin are produced by glial cells closely connecting to neurons and therefore probably being involved in processing of higher brain functions like cognition and memory.

It is hypothesized that abnormal expression of genes involved in structural functions such as cytoskeleton stabilization has a major impact in the pathophysiology of schizophrenia. Therefore, expression of these genes has been studied here on the transcriptional level in the left STG.

## Materials and methods

### Human postmortem tissue

Frozen *postmortem* brain samples from inpatients with DSM-IV residual schizophrenia (*n* = 10) and matched control subjects (*n* = 10) were collected at the Central Institute of Mental Health, Mannheim, and the Department of Neuropathology, Mental Hospital Wiesloch, Germany. Table 1 summarizes the data of patients and controls included in this study. Complete clinical histories were available for all patients. Diagnoses and medical histories were assembled by experienced psychiatrists and are available for all patients in the Wiesloch hospital. Chlorpromazine equivalents (CPE) [30] were used to assess cumulative doses of medication during the last ten years of the patients’ lives. Autopsy consent was obtained from the donor or a family member for each case. All assessments and *postmortem* evaluations and procedures were approved by the Ethics Committee of the Faculty of Medicine, University of Heidelberg, Germany.

**Table 1:** Clinical and demographic data from patients and controls

Code	Diagnosis (2 = co)	DSM IV	Age (years)	Age at onset (years)	Gender	Duration of disease (years)	Hospitalization (years)	Duration of medication (years)	Atyp–typ	CPE last dose (mg)	CPE last 10 years (kg)
01/00	1	295.6	51	23	M	28	17	25	2	450	1.8
13/00	1	295.6	64	16	F	48	21	45	3	1,536	7.7
35/00	1	295.6	64	24	F	41	5	40	2	54.5	4.6
46/00	1	295.6	63	24	F	40	30	30	3	75	1.8
50/01	1	295.6	81	19	M	62	48	50	1	92.8	1.4
83/01	1	295.6	71	30	M	40	12	35	1	782.4	10
36/02	1	295.6	73	30	M	43	33	40	1	507.4	1.7
39/02	1	295.6	43	20	M	22	13	20	3	464	2.6
39/03	1	295.6	77	28	F	49	48	48	2	2,555	8.3
43/03	1	295.6	76	27	F	49	30	47	1	300	4.9
43/01	2		91		F						
61/01	2		66		M						
002/02	2		41		M						
51/02	2		57		M						
57/02	2		53		M						
59/02	2		63		M						
72/02	2		79		M						
RZ77	2		57		M						
RZ84	2		50		M						
RZ99	2		55		F						

Code	Cause of death	PMI (h)	R.I.N. value	Brain Weight (g)	CSF pH	Last medication	Last medication	Cigarettes	Alcohol	ECT
01/00	Heart infarction	12	6.8	1380	6.7	Clozapine 500 mg		30/day	No	No
13/00	Pulmonary insufficiency	11	6.9	1180	7	Clozapine 500 mg	Haloperidol 40 mg, Ciatyl 40 mg	0	No	Yes
35/00	Heart infarction	23	7.6	1250	6.6	Zotepine 150 mg	Olanzapine 10 mg	20/day	No	Yes
46/00	Heart infarction	31	6.1	1240	6.3	Olanzapine 15 mg		30/day	No	Yes
50/01	Cor pulmonale, heart insufficiency	4	6	1390	6.8	Haloperidol 4 mg	Prothipendyl 80 mg	20/day	No	No
83/01	Heart infarction	28	5.9	1480	6.5	Haloperidol 32 mg	Pipamperone 40 mg	40/day	No	No
36/02	Heart infarction	20	7.1	1330	6.9	Perphenazine 32 mg	Promethazine 150 mg	30/day	No	No
39/02	Heart infarction	18	7.8	1520	6.4	Zuclopethixol 40 mg	Valproate 1,200 mg, Tiapride 300 mg	0	No	No
39/03	Lung emboly	32	4.9	1160	6.8	Clozapine 400 mg	Benperidol 25 mg, Chlorprothixen 150 mg	0	No	Yes
43/03	Cardio-pulmonary insufficiency	17	6.1	1200	7.1	Perazine 300 mg		0	No	Yes
43/01	Cardio-pulmonary insufficiency	16	6.1	1120	6.8				No	
61/01	Heart infarction	16	5.7	1200	6.5				No	
002/02	Heart infarction	7	4.7	1360	7.2				No	
51/02	Heart infarction	24	5.5	1420	6.5				No	
57/02	Heart infarction	18	5.5	1520	7.1				No	
59/02	Heart infarction	13	6.6	1360	6.9				No	
72/02	Heart infarction	24	7.8	1170	6.4				No	
RZ77	Electric shock	24	7.6	1380	N.d.				N.d.	
RZ84	Cardiac arrest	50	6.8	1530	N.d.				Yes	
RZ99	Cardiac infarction	14	8	N.d.	N.d.				N.d.	

There were no statistically significant differences between age at time of death, postmortem interval (PMI), and brain pH. Schizophrenia patients were characterized by duration of disease, duration of medication, and medication (last dose) in chlorpromazine equivalents (CPE), as well as cumulative dose over the last ten years in CPE

*SD* standard deviation

Neurovascular or neurodegenerative disorders, such as vascular dementia or Alzheimer’s disease, were excluded by thorough neuropathological examinations [8]. Stagings with respect to neurodegeneration according to Braak were 2 or less for all subjects. Patients and controls had no history of alcohol or drug abuse, or severe physical illness (e.g., carcinoma). Autopsy from controls was performed at the Institute of Neuropathology, University of Heidelberg. Some controls (RZ77, RZ84, and RZ99) were collected by the Neurobiobank, Ludwig-Maximilians-University, Munich. Controls had no history of psychiatric disorders. For patients and healthy controls, gray matter of the left superior temporal cortex (Brodmann area [BA] 22) was dissected by an experienced neuropathologist according to a brain atlas [50], snap-frozen in liquid nitrogen-cooled isopentane, and stored at −80 °C until use.

### RNA preparation and microarray experiments

Total RNA was extracted from dissected snap-frozen tissue using the RNeasy^®^ tissue lipid mini kit (Qiagen), according to the manufacturer’s instructions. RNA concentration and purity was assessed by spectrophotometry (NanoDrop ND1000; NanoDrop Technologies, Delaware, USA). RNA integrity was further assessed using an Agilent 2100 Bioanalyzer and its lab-on-a-chip platform technology (Agilent Technologies UK Ltd, West Lothian, UK). Sample concentrations, 28S/18S ribosomal RNA ratios, and RNA Integrity Numbers (RIN) were automatically calculated with the provided system software [68]. All samples showed RIN values superior to 7.0. Gene expression analysis was performed with the Illumina whole-genome HumanRef8 v2 BeadChip (Illumina, London, UK), covering 24.526 genes with additional splice variants from the RefSeq database. RNA samples were prepared for array analysis using the Illumina TotalPrep™-96 RNA Amplification Kit following the manufacturer’s instructions (Ambion/Applied Biosystems, Warrington, UK). First and second strand cDNA was synthesized from 0.5 μg of total RNA. After dsDNA purification, biotin-labeled cRNA was synthesised. Next, the whole-genome gene expression direct hybridization assay system from Illumina was applied. Samples were loaded on the arrays and assembled into the BeadChip Hyb Chamber. Hybridization was carried out at 58 °C overnight. Subsequently, chips were washed and signals were developed with streptavidin-Cy3. Finally, the BeadChips were scanned using the Illumina BeadArray Reader.

### Data analysis

The data were extracted using BeadStudio 3.2 software (Illumina). Data normalization and gene differential analysis were performed using the Rosetta error model available in the Rosetta Resolver^®^ system (Rosetta Biosoftware) [83]. Fold changes (FC) and P values were generated based on an intensity ratio between control and disease using a conversion pipeline provided by Rosetta. The principal component analysis detected no low quality arrays, and no outliers were detected when conducting a cluster analysis on deregulated genes (*p* < 0.01) using a hierarchical algorithm (agglomerative). A list containing statistically significant, differentially regulated genes with *p* value <0.05 in group-wise *t*-tests was generated. Further cuts were applied based on fold change. These measures resulted in two lists of up-regulated (*n* = 869) and down-regulated (*n* = 896) genes. They were then subjected to an intensity score filtering with a cut-off of 20 fluorescence units (Supplementary Tables S1 [upregulated, 418] and S2 [downregulated, 364]).

### Real-time PCR (qRT-PCR)

For cDNA synthesis, 1 μg of total RNA was reverse transcribed according to the manufacturer’s instructions using the high Cap-Kit from AppliedBiosystems (ABI), Darmstadt, Germany. cDNA was diluted 1:100 before being used as a template. Five microliters of the template was mixed with 5 μl TaqMan GenEx master mix and added to a 384-well plate prespotted with TaqMan probes (spotted in duplicate) for 37 structural genes (for their selection, see Results), including 20 “housekeeping” genes [11] (Supplementary Table S3). The dC_T_ between duplicates was determined, and when dC_T_ > 0.5, the sample was excluded from further analysis. When dC_T_ < 0.5 between duplicates, these values were collapsed as an average for further analysis. The geNorm 3.5 visual basic application (VBA) applet for Microsoft Excel was used to determine the most stable housekeeping genes (http://medgen.ugent.be/~jvdesomp/genorm/) as previously developed and validated by Vandesompele et al. [80]. geNorm determines an expression stability score (M) of a housekeeping gene as the average pairwise variation V for that gene from all the others. All the housekeeping genes are ranked according to their expression stability score using a stepwise exclusion strategy where the housekeeping gene with the worst score is eliminated at each calculation (Supplementary Table S4). The geometric means of the two most stable housekeeping genes (HMBS and RPL27) were used as to normalize expression levels of candidate genes (ddC_T_ method). Data were imported into Statistica 6.1 (StatSoft, Inc., Tulsa, Oklahoma, USA). Differences in transcript abundance between patients and control groups were analyzed using a two-tailed *t*-test for independent samples and were considered statistically significant at *p* < 0.05. Additionally, one-tailed *t*-tests and group-wise tests were performed. The average relative expression level for each group was calculated to determine the FC between groups for each target gene. For further statistical analyses, data were imported in SPSS 17. One way analyses of variance (ANOVA) were performed to evaluate gender effects on gene expressions. Next, for each normalized gene expression, an analysis of covariance (ANCOVA) with factor diagnosis was performed. Covariates age, postmortem interval, and gender were only added to the model, if they had a significant effect in the initial analyses. Pearson’s product moment correlations were calculated to analyze whether normalized gene expressions correlate with age, postmortem interval, duration of disease or hospitalization, age at onset, and medication.

It has to be mentioned that this is an explorative study intending to find differences in the gene expression between schizophrenic patients and control subjects. An adjustment of the error probability would decrease the test power extremely so that the power of detecting existing mean differences would be very low. Therefore, the present results are presented without error probability correction.

### Functional annotation

**Fig. 1:**
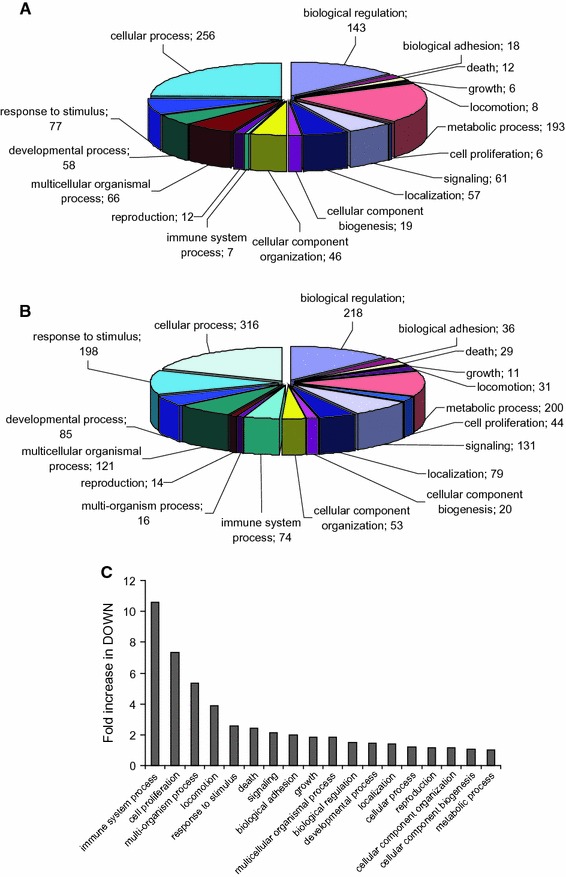
Functional annotation based on Gene Ontology (GO) classification.** a**,** b** Pie charts depicting numbers of up-regulated (A) and down-regulated (B) regulated genes in corresponding Gene Ontology Biological Process layer 2 categories as indicated (see “Methods” for details). **c** Differences between GO layer 2 categories between up-regulated and down-regulated gene sets. Note that the number of genes was higher in the down-regulated set (DOWN) in all categories. Plotted is the relative fold-increase in DOWN to indicate the most prominent changes in the respective processes. Immune-related genes are expressed >tenfold higher in DOWN, cell proliferation >sevenfold und multi-organism processes >fivefold in the down-regulated set

Gene symbols of up- and down-regulated genes were linked to GO classifiers using the utilities/ID converter options of babelomics platform (http://babelomics3.bioinfo.cipf.es/). These lists were uploaded into a locally implemented blast2go annotation software package (http://www.blast2go.com/b2ghome) to analyze GO category distributions at all levels. Biological Process Layer 2 level categories were filtered for minimal 5 members and chosen for visualization (Fig. 1).

## Results

From the lists of 418 up-regulated and 364 down-regulated genes (Tables S1 and S2), which remained after intensity score filtering, 20 up-regulated and 34 down-regulated genes encoding structural gene products (Table S5) (ratio, 0.59) were selected by an experienced investigator. From those, 36 were chosen for qRT-PCR [14 up-regulated and 22 down-regulated (Table 2) (ratio, 0.64)].

**Table 2:** Structural genes chosen for qRT-PCR from differentially regulated genes revealed by microarrays

Struct. Gene Down	Gene ID	*p* value	FC	Struct. Gene Up	Gene ID	*p* value	FC
NM_014979.1	SV2C	0.00371605	−1.70080582	NM_016239.2	MYO15A	0.00379802	1.75784335
NM_004933.2	CDH15	0.00390321	−1.40871141	NM_003763.3	STX16	0.00531162	1.18908491
NM_021019.2	MYL6	0.00812492	−1.28310198	NM_018943.1	TUBA8	0.01092875	1.59036875
NM_001845.3	COL4A1	0.01033501	−1.80484582	NM_025213.1	SPTBN4	0.01339469	1.21306121
NM_175068.2	K6IRS3	0.01825624	−1.40542537	NM_001010925.1	ANKRD19	0.02098038	1.42870811
NM_033401.2	CNTNAP4	0.02201032	−1.78975203	NM_033063.1	MAP6	0.02190119	1.23325508
NM_205848.1	SYT6	0.02211902	−1.28653209	NM_014935.2	PLEKHA6	0.02306862	1.35252326
NM_003280.1	TNNC1	0.02385658	−1.65164908	NM_013231.4	FLRT2	0.02631036	1.35458749
NM_003764.2	STX11	0.02390039	−2.13799226	NM_001856.2	COL16A1	0.02729109	1.34794675
NM_153649.2	TPM3	0.02617573	−1.18259359	NM_080705.2	TRPV1	0.02887878	1.25657791
NM_024933.2	FLJ12056	0.02716962	−1.31591072	NM_145754.2	KIFC2	0.03033599	1.24184565
NM_006571.2	DCTN6	0.02982324	−1.18869974	NM_020405.3	PLXDC1	0.04142866	1.23724641
NM_177424.1	STX12	0.03003602	−1.13874539	NM_145042.2	MGC16703	0.04660599	1.45117471
NM_002404.1	MFAP4	0.03194134	−1.50124258	NM_017514.2	PLXNA3	0.04965694	1.23893469
NM_032639.2	PLEKHA8	0.03339197	−1.69703813				
NM_001855.2	COL15A1	0.03424211	−1.53571389				
NM_007350.2	PHLDA1	0.03702036	−1.38648581				
NM_152403.2	FLJ39155	0.03760172	−1.48267611				
NM_002664.1	PLEK	0.03928154	−1.75702778				
NM_012335.2	MYO1F	0.04072494	−2.57924603				
NM_201281.1	MTMR2	0.04809132	−1.12265023				
NM_006059.2	LAMC3	0.02377526	−1.38905428				

See also Tables S1 and S2

QRT-PCR confirmed 7 of them at the confidence interval of 95 % using the group-wise tests. In the pair-wise tests, 5 genes were significant. Two of them (DCTN6 and TPM3) were not significant in the group-wise tests (Table 3). After lowering the confidence interval to 90 %, 7 more genes were confirmed in the group-wise tests and 5 in the pair-wise tests. It is noticeable that all significant genes resultant from the qRT-PCR were downregulated. In the “trend” group, only one was upregulated in the group-wise tests (MGC16703), but three were upregulated in the pair-wise tests (MGC16703 = tubulin alpha, TUBA8, and KIFC2), which shifts the ratio (0.64) of the genes selected for qRT-PCR (Table 2) to 0.07 and 0.20, respectively, after qRT-PCR (Table 3). It is noticeable that in the two group-wise tests, the significant genes and the non-significant genes are identical, but their ranking is different. Compared to the group-wise tests, in the pair-wise test, only three out of the seven genes significant in the group-wise tests remain significant. However, two genes not significant in the group-wise tests become significant in the pair-wise test (DCTN6 and TPM3). Finally, some of the significant genes in the group-wise tests become non-significant in the pair-wise test (LAMC3, COL4A1, and MYL6), two drop out (COL15A1 and CNTNAP4), and two additional ones appear in the non-significant part of the pair-wise test (KIFC2 and TUBA8). Hence, there are two tubulin genes surfacing in the pair-wise test. The significance levels of the last four genes in the pair-wise test are below 90 % confidence interval and are only shown for comparative purpose with the group-wise tests.

**Table 3:** QRT-PCR results showing significantly regulated structural genes (*bold*) and genes that did not quite reach significance level

Gene symbol	Gene name	*p* value (2)	Gene symbol	*p* value (1)	Gene symbol	*p* value (pw)
**MTMR2**	Myotubularin-related protein 2	0.007121447	**COL15A1**	0.007070113	**STX12**	0.000514191
**LAMC3**	Laminin gamma 3	0.007877202	**STX12**	0.013457459	**MTMR2**	0.017657646
**SYT6**	Synaptotagmin 6	0.010554885	**MTMR2**	0.014776572	**SYT6**	0.018046627
**COL15A1**	Collagen XV, alpha 1	0.010614934	**SYT6**	0.018015277	**DCTN6**	0.040844042
**STX12**	Syntaxin 12	0.011662093	**LAMC3**	0.023460519	**TPM3**	0.042311935
**COL4A1**	Collagen IV, alpha 1	0.041537481	**COL4A1**	0.039728237	LAMC3	0.062943756
**MYL6**	Myosin, light chain 6	0.050687473	**MYL6**	0.04887248	MFAP4	0.068474312
PLEK	Pleckstrin	0.076886961	PLEK	0.07730228	KIFC2 up	0.085389609
CNTNAP4	Contactin-associated protein 4	0.089501846	MFAP4	0.083516435	TUBA8 up	0.104664969
MFAP4	Microfibril-associated protein 4	0.0901955	CNTNAP4	0.094026743	FLJ12056	0.104863175
DCTN6	Dynactin 6	0.092758551	DCTN6	0.105156826	MGC16703 up	0.11362909
FLJ12056	Ankyrin repeat domain 53 (ANKRD53)	0.102586666	MGC16703 up	0.105301982	COL4A1	0.156740231
TPM3	Tropomyosin 3, variant 2	0.108070257	TPM3	0.106877524	PLEK	0.174848002
MGC16703 up	Tubulin alpha, pseudo	0.108094601	FLJ12056	0.217773003	MYL6	0.190495998
		(2) group-wise two-tailed		(1) group-wise one-tailed		(pw) pair-wise

Three tests have been performed: two-tailed (2), one-tailed (1) group-wise, and pair-wise (pw) *t*-tests. Note that downregulated genes are overrepresented compared to genes from Table 2 and that some genes not significant in group-wise tests are significant in pair-wise tests

### Potential confounds

Patients did not differ from controls regarding age, postmortem interval (PMI), pH of CSF, and RIN values. Controls consisted of 8 men and 2 women, and schizophrenia patients were 5 men and 5 women. Therefore, the influence of gender on the results was calculated. The 36 genes included in qRT-PCR were subjected to stepwise regression and ANCOVA as described above. No influence on the expression of the nine significant structural genes was found by (i) gender, (ii) age of onset, (iii) medication in chlorpromazine equivalents (CPE) last dose and cumulative dose during the last 10 years, (iv) duration of medication, and (v) disease duration. An immunohistochemistry study also did not find changes in various synapse-associated proteins in rat brain under treatments with perphenazine, chlorpromazine, trifluoperazine, or haloperidol [62].

## Discussion

### General remarks

**Fig. 2:**
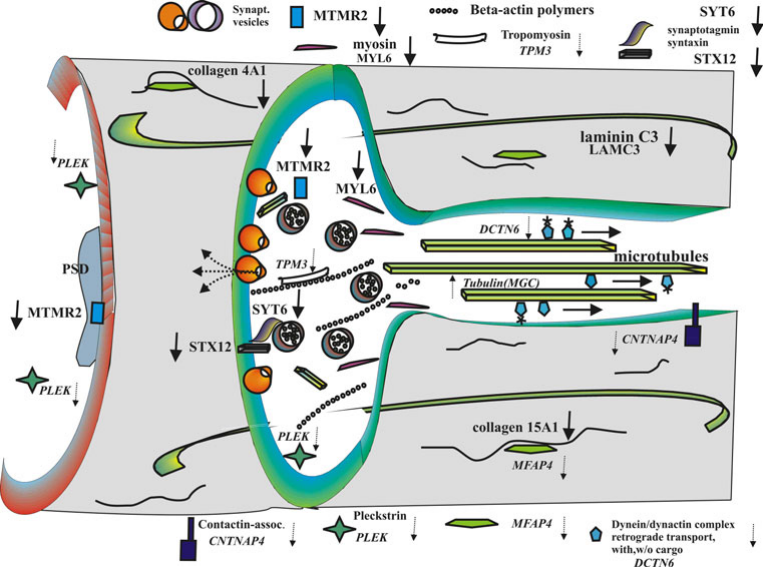
Differentially regulated extra- and intra-cellular structural elements at the synapse. Significantly regulated genes and their regulation by *arrows* are in *bold* (see Table 3). *Shaded area* is extracellular matrix. For more details, see “Discussion”

Presynaptic terminals are highly specialized and hierarchized structures composed of cytoskeletal and membrane elements elaborated to organize fine-tuned exo- and endo-cytosis of synaptic vesicles. Neurotransmitter release closely relies on the local transport of vesicles by microfilaments and attachment of vesicles to actin filaments by synapsins [4, 31]. Recently, 410 proteins have been reported to be associated with synaptic vesicles and presynaptic membranes [77]. Synaptic components are transported along the length of axons on microtubules loaded on motor proteins of the kinesin-3 family [54]. Nearly, all aspects of synapse structure and function are in a constant state of flux resulting in synapse stabilization or elimination [47, 49, 74, 79] (Fig. 2).

A new finding reveals that synapse elimination involves molecular cascades traditionally known from the innate immune system [63]. In a recent microarray study of the left STG of the same patients as investigated in this study, we found decreased expression of immune-related genes in schizophrenia, discussing their impact on synaptic function [66]. In the present microarray study, we also found downregulation of brain-derived neurotrophic factor (BDNF). This neurotrophin regulates synaptic plasticity, and a lack of BDNF results in alterations of excitatory and inhibitory synapses [15]. Moreover, its Val66Met gene polymorphism has been suggested to be involved in the pathophysiology of schizophrenia, with particular emphasis on dysfunctions of the hippocampus [21].

### Altered synapse-specific components

#### Synaptotagmin 6 (SYT6) (down)

SYTs are known to operate by binding to calcium ions, anionic lipids, and to syntaxin (STX). Three distinct kinetic groups of SYTs have been described: SYT1–3, a fast group; SYT5, 6, 9, 10, a medium group, and SYT7, the group showing the slowest kinetics. The medium group is believed to function as calcium sensors for asynchronous release [19]. In our study, SYT6 was found to be downregulated in the left STG of schizophrenia patients. As a calcium sensor [44], it triggers exocytosis of presynaptic vesicles. Because it is a component of the asynchronous or delayed release, the duration of its action persists for longer times (10–100 ms after collapse of calcium domains). Influenced by the calcium-synaptotagmin complex, synaptobrevin and syntaxin form a type of ion channel that opens to form the fusion pore [24]. Reduced expression of SYT6, hence, attenuates calcium-triggered neurotransmitter release. The deficits here observed in schizophrenia patients could compromise phasic release.

#### Syntaxin 12 (STX12) (down)

Presynaptic vesicle proteins like synaptosome-associated protein 25 (SNAP-25) and syntaxin form the soluble N-ethylmaleimide-sensitive factor attachment protein receptor (SNARE) complex. In the anterior frontal cortex, syntaxin 1 has been reported to be decreased in schizophrenia [27]. Other members of the syntaxin group have not been investigated so far in this context. The major function of syntaxins appears to be their direct involvement in transpairing between synaptobrevin and syntaxin, which physically forces the synaptic vesicle membrane and the presynaptic membrane into contact, a final step of synaptic vesicle exocytosis.

Syntaxin 12 shows a strong localization with the endosome [78]. It modifies the affinity of the glutamate receptor GLUR2 and of the glutamate receptor interacting protein1 (GRIP1) [75] with the endosomal protein neuron-enriched endosomal protein of 21kDa (NEEP21) and consequently GLUR2 recycling. Along these lines, SYT12/13 has been shown to be involved in recycling of various other receptors and also in neurite outgrowth [58]. Therefore, downregulation of STX12 in the STG of schizophrenia patients implies a subtle impairment of synaptogenesis.

### Cytoskeletal components

#### Myosin light chain 6 (MYL6) (down)

Actin–myosin interactions are known to regulate dendritic spine shape. Myosins induce the formation of short, mushroom-shaped spines by enhancing actomyosin contractility or lead to spine stabilization through gelsolin capping activity [34]. Myosins can also interact with NR1 and NR2 subunits of NMDA receptors [53]. The recently discovered coiled-coil protein associated with myosin 2 and DISC1 (CAMDI) interacts with disrupted-in-schizophrenia-1 (DISC1) and influences radial migration [16]. Therefore, myosins are possibly involved in the pathophysiology of schizophrenia. Specifically, the myosin 2, 5, and 6 isoforms were found in dendritic spines, and their downregulation may alter spine shape and synaptic plasticity in schizophrenia.

#### Pleckstrin (PLEK) (down)

The reorganization of the actin cytoskeleton with subsequent changes of cellular morphology and cell spreading is highly dependent on phosphorylated pleckstrin [28, 39]. Pleckstrin has been associated with schizophrenia (linked to a catechol-o-methyltransferase Val108/158 Met functional polymorphism), and Sei et al. [69] have recently reported that neuregulin1-ErB signaling is PI3K/Akt/Pleckstrin dependent and impaired in patients with schizophrenia.

#### Tropomyosin 3, variant 2 (TPM3) (down)

Tropomyosin (Tm) isoforms, integral components of actin microfilaments, form coiled-coil head-to-tail dimers that bind actin polymers [55]. In vitro studies have implicated Tms in the stabilization of the actin cytoskeleton by protecting actin filaments from the severing action of gelsolin [29] and the depolymerising action of ADF/cofilin [3]. Decreased expression of the high-molecular-weight tropomyosin isoform Tm3 (αTM*slow*, = tropomyosin 3/*TPM3*) in schizophrenia may result in an initial inhibition of neurite outgrowth followed by a significant decrease in the number and length of dendrites [64]. In a proteomic study of the left STG in the same cohort of schizophrenia patients and controls, Martins-de-Souza et al. [42] found downregulation of tropomyosin alpha 3 chain, confirming our results on gene expression.

#### Tubulin alpha pseudogene (MGC16703) and tubulin alpha (up)

Microtubules are composed of alpha-tubulin and beta-tubulin heterodimers and are known to play a vital role in numerous cellular processes including intracellular trafficking, migration, and mitosis [33]. In adulthood, *Tuba1a* is expressed in neurons of the olfactory bulb, the rostral migratory stream, and the dentate gyrus [10]. Its role in hippocampal neurogenesis has been proposed recently [32]. To date, its specific role in the pathophysiology of schizophrenia is unknown. Our results are confirmed by a recent proteomic investigation of the same region in the same cohort of schizophrenia patients showing downregulation of tubulin beta-5 chain, tubulin alpha-1 chain, and tubulin alpha-ubiquitous chain, whereas tubulin beta-3 chain was upregulated [42]. The upregulation of tubulin alpha as shown in our study may be a compensatory reaction to altered protein expression.

#### Myotubularin-related protein 2 (MTMR2) (down)

Myotubularin-related protein 2 belongs to the families of phosphotyrosine phosphatase/dual specificity phosphatase (PTP/DSP), and its likely physiological substrate is phosphatidylinositol (3,5) bisphosphate, a key regulator of vesicle transport [51]. Mutations in MTMR2 cause the autosomal recessive Charcot–Marie–Tooth (CMT)-type 4B1 demyelinating neuropathy [36], which is characterized by reduced nerve conduction velocity and focally folded myelin sheets in peripheral nerves [59]. The substrate specificity of active MTMRs suggests a function in endocytosis, sorting, and degradation of proteins in early or late phases of endocytosis. Loss of function produces abnormalities in cell adhesion and cell polarity [6]. Along these lines, it has been found to interact with the membrane-associated guanylate kinase-like (MAGUK) protein complex, which is typically localized in postsynaptic densities of central neurons [5]. Interestingly, it has been shown that MTMR2 requires a Pleckstrin homology-GRAM domain for membrane association [2], and both MTMR2 and PLEK have been found here to be downregulated in the STG of schizophrenia patients.

#### Dynactin 6 (DCTN6) (down)

Dynactin plays a role in regulating or coordinating the functions of dynein, which is responsible for a variety of microtubule-based movements of vesicles and organelles [40]. The final process of terminal branching, synaptogenesis, and stabilization of sensory axons requires the dynein–dynactin complex [48]. In the pathophysiology of schizophrenia, aberrant synaptic trafficking of endosomal vesicles has been suggested [61]. In this context, the dynactin complex has been reported to interact with dysbindin and disrupted-in-schizophrenia-1 (DISC1), both of them being confirmed as risk genes of schizophrenia [43].

### Components of the extracellular matrix

#### Laminin gamma 3 (LAMC3) (down)

Formation and maintenance of synapses in the mammalian nervous system is critically dependent on extracellular matrix (ECM) molecules*.* Laminin has been implicated in the morphogenesis of the nervous system due to its increased expression along CNS developing pathways [46] and its ability to promote cell migration, differentiation, and axonal guidance. Laminins are produced mainly in glia cells [57], but also in neurons [22]. The specific function of the gamma3 chain in synapse formation is unknown. We are here the first to report decreased expression of laminin in the STG in schizophrenia. In the parieto-occipital cortex, increased laminin protein has been reported along with no alterations on the mRNA level [35].

#### Collagens XV alpha 1 and IV alpha 1 (COL15A1, COL4A1) (down)

Developmental expression of different isoforms of collagen ensures the precise maturation and proper function of a synapse throughout different phases of a neuron. Therefore, it comes of no surprise that disturbances of collagen expression is related to severe disorders, such as HANAC (Hereditary Angiopathy, Nephropathy, Aneurysms, and Cramps) syndrome, a COL4A1-related disorder that presents with retinal tortuosity and muscle cramps and with variable combinations of small vessel brain disease [56], or the “COL4A1 stroke syndrome” [81]. Furthermore, COL4A1 malfunction has been associated with cerebral microangiopathy, the Axenfeld-Rieger anomaly, and leukoencephalopathy and stroke [71]. Collagen XV is pivotal to peripheral nerve maturation. Lack of collagen XV and laminin α4 leads to severely impaired radial sorting and myelination, compromising the maturation of peripheral nerves [60]. Furthermore, several other forms of collagen accumulate at the synaptic cleft. In a recent study, it was reported that col19a1, the gene encoding non-fibrillar collagen XIX, is expressed by subsets of hippocampal neurons. These subsets of synaptotagmin 2 (Syt2)-containing hippocampal interneurons (neuropeptide Y (NPY)-, somatostatin (Som)-, and calbindin (Calb)-immunoreactive interneurons) with inhibitory synapses appear malformed in the absence of collagen XIX [76]. Therefore, downregulation of collagens is in line with a dysfunction of GABAergic interneurons in schizophrenia [9, 17].

#### Microfibril-associated protein 4 (MFAP4) (down)

Microfibril-associated protein 4 is a collagen-binding protein that resides in the extracellular matrix in association with elastic fibers and binds to the collagen-like domain of surfactant protein A (SP-A). Moreover, it binds in a calcium-dependent manner to gelatine and the collagen-like domain of SP-D [37]. The Marfan-syndrome, a disease of microfobril dysfunction, has been associated with schizophrenia in multiple case reports [38]. To date, we are the first to report downregulation of MFAP4 in schizophrenia.

### Axon-associated component: relevance for schizophrenia

#### Contactin-associated protein-like 4 (CNTNAP4) (down)

Contactin-associated protein is uniquely expressed in the nervous system and found together with contactin at the paranodal junctions between axons and the terminal loops of oligodendrocytes. It is a transmembrane protein, displaying high structural similarity with neurexins. [45]. Its downregulation is in line with the hypothesis of dysconnectivity based on dysfunctional oligodendrocyte-neuronal interactions in schizophrenia [65, 67].

## Limitations

One limitation of the study is the false discovery rate contained in the microarray data, likely to be carried over to qRT-PCR evaluations. This implies that the risk of false positive findings cannot be neglected, especially in the microarray analysis, where a large number of genes (>24,000) was analyzed, which raises problems for multiple testing. However, when taking into consideration that a mental disorder like schizophrenia is caused by multiple genes and the contribution of each single gene is small, increasing the statistical stringency automatically entails a loss of biological information. It is possible that the statistically most significant genes are not the most important ones on a functional level considering an interplay of genes in complex pathways. Admittedly, the study should be confirmed in an independent sample.

## Conclusions

Our results of several differentially expressed genes in the left STG confirm that schizophrenia is a multifactorial disorder leading to alterations in several disease-related pathways (Fig. 2). All genes confirmed by qRT-PCR to be significantly regulated were downregulated. This strongly supports the notion of a general weakening of synaptic strength, disturbances of connectivity, and subsequent axon retraction in schizophrenia. It is well in accordance with the morphological findings of volume loss in the STG. The results, however, do not reveal whether or not the downregulation of structural elements prefers certain types of neurons, for example, glutamatergic or allow predictions about the course of events, that is, primary destabilization of ECM elements followed by depolymerization of actin and degradation of myosin and tubulin. Moreover, the results do not provide evidence of the driving forces of these downregulations, which could be changes in transcription factor activities but also epigenetic changes on DNA promoter or intron sequences. To obtain some idea of these mechanisms, it is necessary to investigate younger populations of schizophrenia patients and animal models of risk factors of schizophrenia during brain development [73].

## Electronic supplementary material

Below is the link to the electronic supplementary material.

Supplementary material 1 (XLS 94 kb)

Supplementary material 2 (XLS 85 kb)

(JPG 303kb)

(JPG 405kb)

(JPG 1266kb)
