# Supplementary figures and images for: Structural synaptic elements are differentially regulated in superior temporal cortex of schizophrenia patients
Source: Eur Arch Psychiatry Clin Neurosci. 2012 Mar 23;262(7):565–77. doi: 10.1007/s00406-012-0306-y (PMC3464383; doi:10.1007/s00406-012-0306-y)

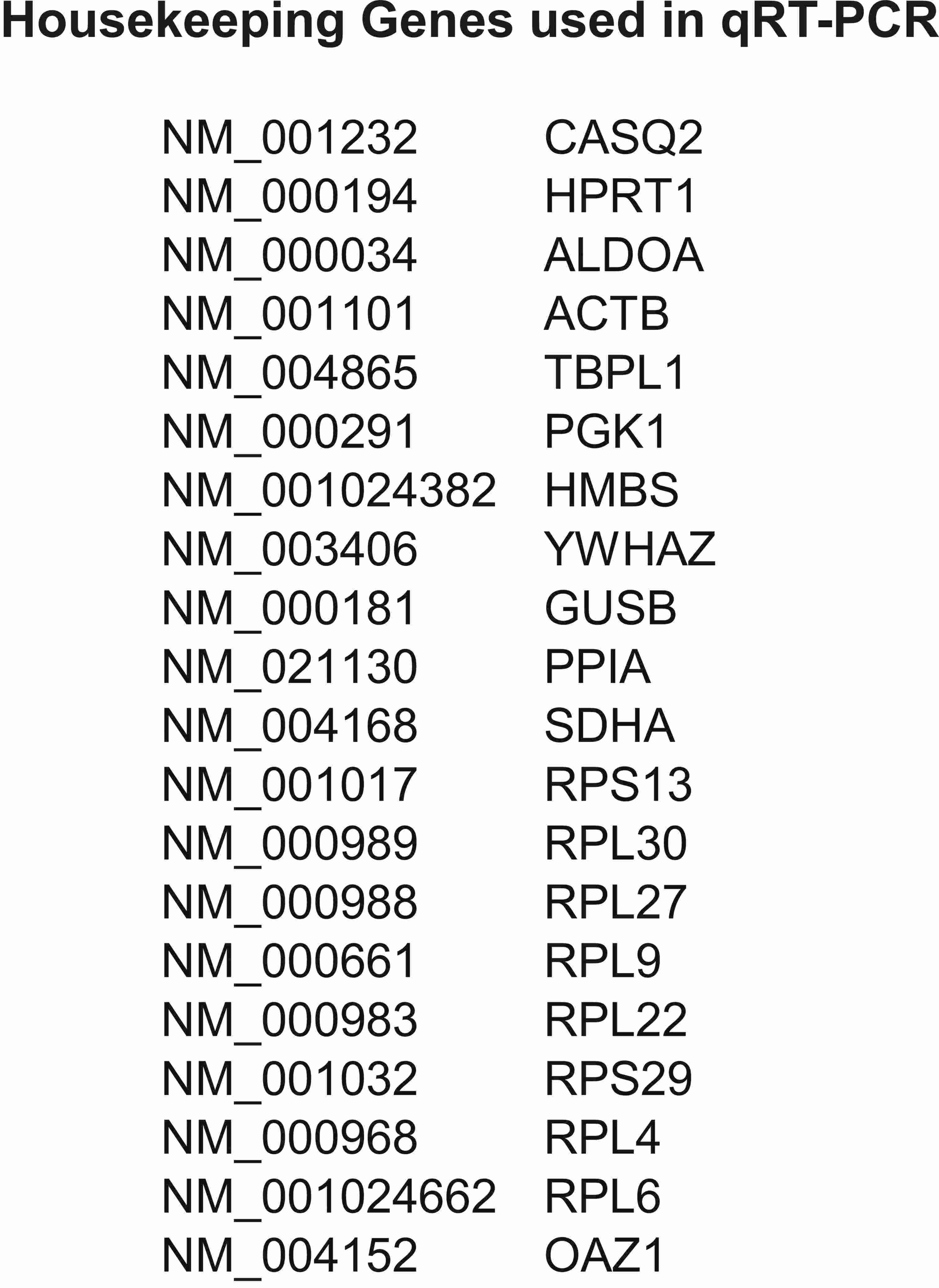

Supplement: Supplementary file 3 — (JPG 303kb) [file 406_2012_306_MOESM3_ESM.jpg]

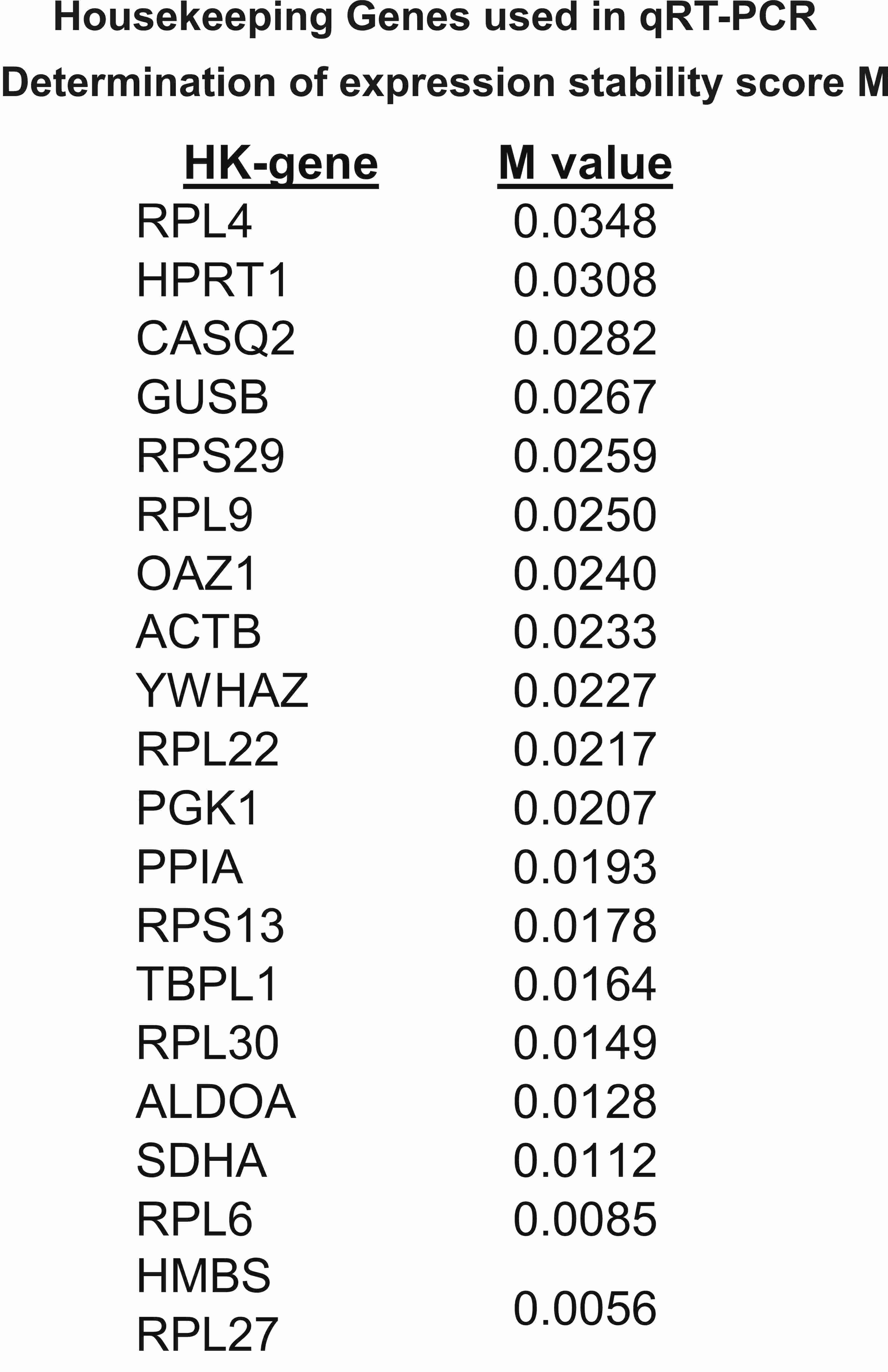

Supplement: Supplementary file 4 — (JPG 405kb) [file 406_2012_306_MOESM4_ESM.jpg]

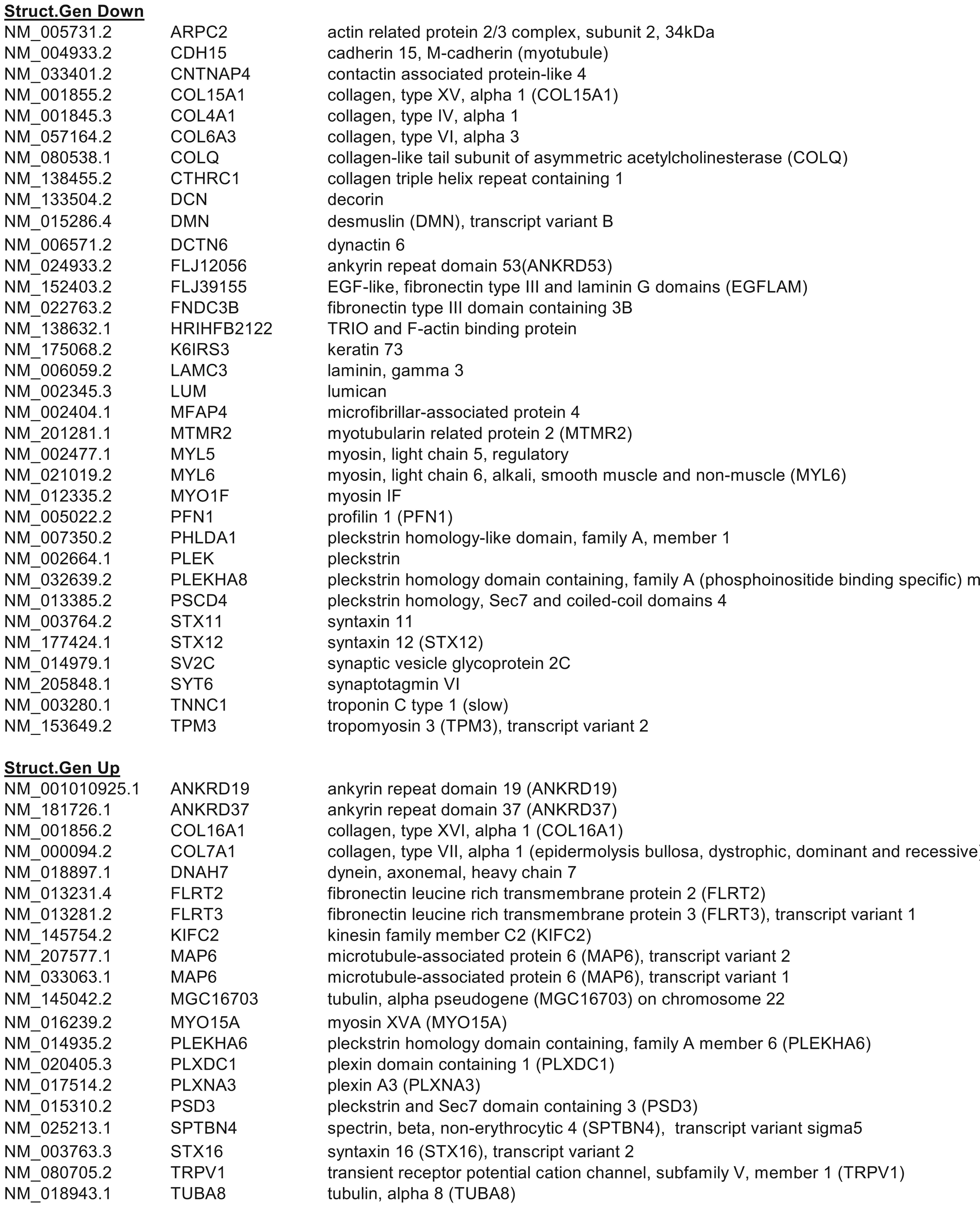

Supplement: Supplementary file 5 — (JPG 1266kb) [file 406_2012_306_MOESM5_ESM.jpg]
